# Association between chronic diseases in childhood and subsequent educational achievement: a Danish register-based cohort study

**DOI:** 10.1007/s10654-025-01315-9

**Published:** 2025-10-14

**Authors:** Ann-Sophie Buchardt, Andreas Jensen, Helene Kildegaard, Lone Graff Stensballe

**Affiliations:** 1 Mary Elizabeth’s Hospital, Rigshospitalet, Copenhagen University Hospital, Copenhagen, Denmark; 2 Department of Paediatrics and Adolescent Medicine, Rigshospitalet, Copenhagen University Hospital, Copenhagen, Denmark; 3 Department of Clinical Medicine, University of Copenhagen, Copenhagen, Denmark

**Keywords:** Severe chronic disease, Education, School-aged-children, Population-based

## Abstract

Severe chronic disease (SCD) in childhood may hinder not only physical health but also academic performance. In this population-based cohort study, we investigated educational outcomes among 20,979 Danish children with SCD (54.7% male) and 423,814 without SCD (51.1% male). We assessed completion of lower secondary school and grade point averages (GPAs). Completion and GPAs with 95% confidence intervals (95% CI) were estimated using logistic and linear regression models adjusted for sex, country of origin, and maternal education. Children with SCD had lower probability of completing 9th grade (male: 0.53 [95% CI 0.52$-$0.54], female: 0.63 [0.62$-$0.64]) than their peers without SCD (male: 0.70 [0.70$-$0.70], female: 0.82 [0.81$-$0.82]). Similarly, GPA was lower for children with SCD (male: 6.61 [6.55$-$6.66], female: 7.51 [7.45$-$7.56]) compared to those without (male: 6.86 [6.85$-$6.87], female: 7.90 [7.89$-$7.91]). The sex disparity persisted across all groups. Children of mothers with lower education experienced larger performance gaps. Neurological and perinatal conditions showed the poorest outcomes. Our findings demonstrate persistent educational inequalities among children with SCD, even in settings with universal healthcare and education, underscoring the need for targeted, cross-sectoral support.

## Introduction

Chronic diseases in children present significant challenges, not only to their physical health but also to their social development, emotional well-being, and educational attainment [1, 2]. Frequent hospital visits, complex treatments, and persistent symptoms such as pain and fatigue may lead to school absenteeism and disengagement, placing children with severe chronic disease (SCD) at risk of falling behind academically [2–4]. Poor school performance and lower educational attainment are, in turn, associated with adverse adult outcomes, including poorer employment prospects, lower income, and reduced health [5–9]. The impact of chronic illness on academic achievement varies considerably depending on the nature and severity of the underlying condition [10, 11]. No consistent associations with academic performance have been found for asthma [4], while evidence for type 1 diabetes remains conflicting [12, 13]; in contrast, childhood cancer and epilepsy are generally associated with poorer academic achievement, though the extent varies by cancer type [4, 14, 15]. Several mechanisms may explain these disparities. For instance, children with neurological conditions, such as spina bifida or epilepsy, may experience cognitive impairments that directly hinder academic performance, while others, such as children undergoing cancer treatment, may struggle primarily with side effects to medical treatment and prolonged absences [2]. Beyond disease-specific effects, socioeconomic context, parental support, and school resources may further contribute to disparities in academic outcomes [16–19]. Despite growing evidence, most studies to date have focused on single conditions or have relied on surveys or clinical cohorts, predominantly from North American settings [9, 10, 20, 21]. These studies may not generalize to many European countries with universal health and educations systems. In addition, only few studies have examined educational outcomes across wider spectra of chronic disorders using large-scale, population-based data [11, 17]. This study aimed to examine educational outcomes among children with SCD in Denmark using nationwide register data. Specifically, we assessed the risk of not completing primary and lower secondary school and the grade point average among those who completed all final exams. We further explored variation in academic performance across diagnostic subgroups and sociodemographic characteristics to inform targeted educational and clinical interventions.

## Methods

### Study design and data source

This is a population-based cohort study. Data were extracted from various Danish national registers, including health registers [22] and Statistics Denmark [23], covering data on mortality, socioeconomic variables, and migration. The Central Person Registration (CPR) [24] ensured linkage of data at an individual level. Key data sources included: the Danish Medical Birth Register (MBR; [25]), the Population Register [24], the Migration Register [24], the Danish National Patient Register (DNPR; [26]), and several Education Registers [27] including data from the Danish National Test program (DNT), which are covered in "Appendix A".

### Study population

The study population included all live-born children born in Denmark between 2000 and 2006 (both included) who resided in Denmark 31 July the year they turned six (without prior migration). We excluded individuals who had an unknown country of origin. Here, country of origin was defined as parental country of origin meaning that the they had a mother and/or father who was born in Denmark. We also excluded individuals if the mother of the individual was not alive 31 July of the year the individual turned six. While it would be possible to use the mother’s educational level at the time of death to avoid missing data, we believe that losing a mother is fundamentally different from having a living mother with a certain educational level.

### Exposure

Severe chronic disease (SCD) in children was identified using the International Classification of Diseases, Tenth Revision (ICD-10) ICD-10 codes, as outlined in "Appendix B". This definition was developed by clinicians based on diagnoses considered potentially life-threatening [28, 29]. The same definition has been applied in previous research and has been shown to be strongly associated with mortality among children in Denmark [30]. Children with SCD were identified in the DNPR using specific diagnosis codes registered in relation to hospital contacts. To estimate the effects of the inherently time-dependent covariates SCD and maternal educational level, we used a landmark approach. The primary landmark was set at 31 July of the year the child turned six. A clinical diagnosis of an SCD from birth to the landmark point defined the exposure status. In the following, this is referred to as“SCD diagnosis”(yes/no).

### Outcomes

Most children in Denmark begin primary school in the year they turn six and complete it after approximately 10 years (grades 0–9). Primary and lower secondary school is completed during the final year (9th grade) after attending eight mandatory exams: four Danish exams (reading, writing, spelling and oral), two written mathematics exams (one with and one without aids), one oral English exam and one oral science exam, along with two randomly selected exams (e.g., German or history). In addition, a mandatory project is required in the 8th grade. The exams are graded using a seven-point scale: -3, 0, 2, 4, 7, 10, and 12 [31].

We included two co-primary outcomes to evaluate school performance in children with and without SCD. First, we examined completion of lower secondary school before September the year of turning 17 years. This was defined as attending all eight final mandatory exams in the 9th grade with a weighted grade point average (GPA) above 2.0 (as defined by the completion criteria set forth as of 2017). Second, we analyzed the achieved weighted GPAs of the children who attended all eight mandatory exams. We analyzed the overall weighted GPA for all exams and for the Danish and mathematics exams separately to investigate skills representing different cognitive functions. Furthermore, we examined the associations between potential risk factors and the inability to complete lower secondary school in children with SCD.

Children enrolled in Danish public schools are required to follow The Danish National Test Program (DNT) during compulsory schooling. Using data from this source, we included two co-secondary outcomes to evaluate repeated measures of school performance in children with and without SCD. We examined the results from the Danish/reading tests in grades 2, 4, 6, and 8 and from the mathematics tests in grades 3, 6, and 8 ("Appendix A").

### Covariates

To examine performance according to demographic and socioeconomic characteristics, we included information on the child’s sex (assigned at birth), country of origin (Danish or not), and maternal education. Maternal education was defined as the highest level of formal education completed by the mother on 31 July of the year the child turned six, categorized according to the UNESCO international standard classification of education [32] and divided into four groups: primary education, secondary education, bachelor’s degree, and master’s or PhD level. For details see "Appendix C".

### Statistical methods

The completion of 9th grade was analyzed using logistic regression, and associations were presented as probabilities with 95% prediction intervals ("Appendix C"). The analyses were conducted on the full study population ($N = 444,793$). The grade point average (GPA) in 9th grade was analyzed using linear regression. This analysis was performed on a subpopulation ($N = 410,540$) conditional on the individual having attended 9th grade exams by 30 June of the year they turned 17. Both the estimated probability of completing 9th grade and the estimated GPAs at exams in 9th grade were based on three versions of the main model: one overall model of the exposure effect on the outcome, three marginal interaction models of the exposure effect on the outcome adjusted for sex, country of origin, and maternal education, respectively, and one expanded model adjusted for both sex, country of origin and maternal education, that is, including an interaction term between SCD and each of the covariates. Comparisons of the difference in effect of levels of sex, country of origin, and maternal education within the exposure groups were presented as relative risks (the ratio of the probability of completing of 9th grade with SCD to the probability of completing of 9th grade without SCD) and mean differences for completion of 9th grade and GPA in 9th grade, respectively. For the Danish National Tests ($N=155,520$), combined scores across the three cognitive domains from Danish/reading tests in grades 2, 4, 6, and 8 and mathematics tests in grades 3, 6, and 8 were analyzed using mixed-effects models ("Appendix A").

### Supplementary analyses

Supplementary analyses were performed for the two co-primary outcomes, completion of grade 9 and grade 9 GPA.

Subject-specific GPA analyses were performed to study variation in educational achievement between the subjects Danish and mathematics.

Subgroup analyses by type of chronic disease were performed to explore variation in educational achievement across specific groups of diagnoses. For this, diagnoses were categorized according to organ system in the ICD-10 ("Appendix B"). For each subgroup analysis, individuals were classified into one of three groups: (1) the specific SCD group being analyzed, (2) individuals with other types of SCD, and (3) individuals with no SCD diagnosis.

Further, to assess the effect and robustness of the landmark approach, we repeated the analyses across a grid of landmark ages. Specifically, we updated the information on SCD status, maternal education, migration, and death annually by 31 July, from the year of turning one to the year of turning 16, thereby obtaining point wise estimates and 95% confidence intervals.

Data management and statistical analyses were performed using R version 4.4.1 [33]. The mixed effects linear regression models were implemented using the lme4 package [34].

## Results

**Fig. 1:**
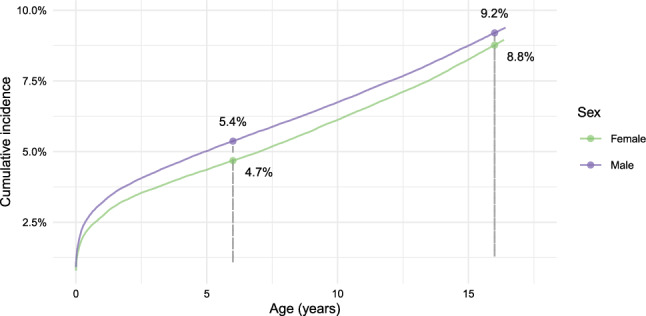
Percentage of the population at risk with at least one SCD diagnosis (2000–2023). Children and adolescents were followed from birth until first severe chronic disease (SCD) diagnosis, death, migration, or the last follow-up date (30 June the year of turning 17)

Characteristics of covariates and outcomes across the study populations used in the two co-primary analyses are provided in Table 1. Among the 457, 674 children born in Denmark between 1 January 2000 and 31 December 2006 a total of 444,793 were included in the first co-primary analysis of which 20,979 (4.7%) had an SCD diagnosis before 31 July the year they turned six. At age 6, the cumulative incidence of SCD was higher in boys (5.4%) compared to girls (4.7%) (Fig. 1). We excluded 2,437 (0.5%) children who died, 9,369 (2%) children who migrated before the primary landmark, 346 (0.1%) children with an unknown country of origin, and 759 (0.2%) children whose mother died before the primary landmark. Among the included children, 216,891 (48.8%) were female, 399,380 (89.8%) had at least one parent of Danish origin, and 178,703 (40.2%) were born to mothers whose highest level of education was upper secondary school, the most common level of maternal education at the primary landmark. Covariate distributions are provided in the "Appendix D". Among the children and adolescents included in the study, 379 (0.1%) died and 1, 915 (0.4%) migrated before 30 June the year of turning 17.

**Table 1:** Characteristics (observation counts and mean grade point averages [GPAs]) of the study populations used for the analyses of completion 9th grade and GPA in the 9th grade

	Completion of grade 9		Grade point average	
Characteristic	No. (%)	Comp. (%)	No. (%)	Mean (SD)
**Overall**	444,793	332,295 (74.7%)	410,465	7.4 (2.6)
*Severe chronic disease*				
No	423,814 (95.3%)	320,191 (75.5%)	393,615 (95.9%)	7.4 (2.6)
Yes	20,979 (4.7%)	12,104 (57.7%)	16,850 (4.1%)	7.0 (2.7)
Neoplasms	386 (0.1%)	235 (60.9%)	338 (0.1%)	7.3 (2.6)
Blood	213 (0.0%)	125 (58.7%)	182 (0.0%)	6.7 (2.8)
Endocrine	946 (0.2%)	631 (66.7%)	812 (0.2%)	7.2 (2.7)
Nervous system	3,524 (0.8%)	1,542 (43.8%)	2,394 (0.6%)	6.7 (2.8)
Circulatory	634 (0.1%)	415 (65.5%)	541 (0.1%)	7.4 (2.8)
Respiratory	614 (0.1%)	402 (65.5%)	554 (0.1%)	6.6 (2.8)
Digestive	2,050 (0.5%)	1,436 (70.0%)	1,833 (0.4%)	7.3 (2.7)
Musculoskeletal	1,398 (0.3%)	924 (66.1%)	1,230 (0.3%)	7.2 (2.7)
Genitourinary	461 (0.1%)	309 (67.0%)	404 (0.1%)	7.1 (2.8)
Perinatal	1,056 (0.2%)	429 (40.6%)	757 (0.2%)	6.8 (2.7)
Malformations	9,697 (2.2%)	5,656 (58.3%)	7,805 (1.9%)	7.0 (2.7)
*Sex*				
Male	227,902 (51.2%)	157,160 (69.0%)	208,324 (50.8%)	6.9 (2.6)
Female	216,891 (48.8%)	175,135 (80.7%)	202,141 (49.2%)	7.9 (2.5)
*Country of origin*				
Denmark	399,380 (89.8%)	304,534 (76.3%)	373,275 (90.9%)	7.5 (2.6)
Not Denmark	45,413 (10.2%)	27,761 (61.1%)	37,190 (9.1%)	6.3 (2.7)
*Maternal education*				
Primary	74,174 (16.7%)	43,169 (58.2%)	64,039(15.6%)	5.8 (2.7)
Upper secondary	178,703 (40.2%)	133,820 (74.9%)	166,795 (40.6%)	7.0 (2.6)
Bachelor’s	136,650 (30.7%)	111,324 (81.5%)	129,393 (31.5%)	8.1 (2.3)
Master’s or PhD	48,208 (10.8%)	40,595 (84.2%)	45,419 (11.1%)	9.1 (2.0)
Unknown	7,058 (1.6%)	3,387 (48.0%)	4,819 (1.2%)	6.0 (2.7)

### Completion of 9th grade

**Fig. 2:**
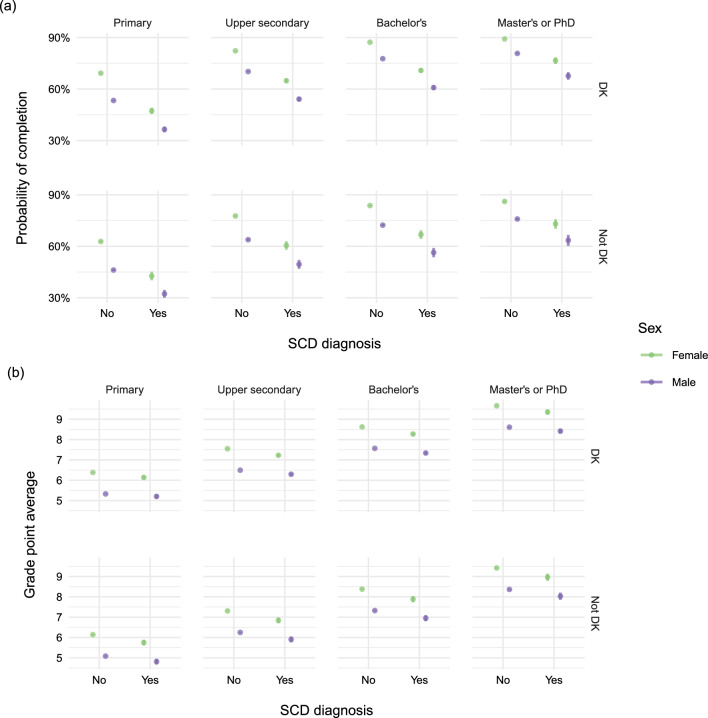
**a** Estimated probability of completing 9th grade, and **b** estimated grade points average in 9th grade. Results were adjusted for sex, severe chronic disease (SCD) diagnosis, country of origin, and maternal education

In the overall analysis of completing 9th grade, we found that individuals with an SCD diagnosis had 76% lower probability of completing 9th grade compared to adolescents without an SCD diagnosis. This pattern was observed in the marginal analysis with interactions (Table 2) and in the expanded analysis including all covariates when comparing adolescents with same sex, country of origin, and maternal education. The estimated probabilities across all combinations of covariates are visualized in Fig. 2a. Both non-Danish country of origin and lower maternal education were associated with lower probability of completing 9th grade as seen in Fig. 2a.

**Table 2:** Relative risks of completion of 9th grade and mean differences of grade point averages (GPAs) between individuals with SCD (yes) and without SCD (No) with 95% confidence intervals from marginal interaction models of the exposure effect on the outcome adjusted for sex, country of origin, and maternal education, respectively

Categories	Relative risk	Mean difference
	Yes / No	Yes - No
*Severe chronic disease*		
Overall	0.76 (0.75,0.77)	$-$0.36 ($-$0.40,$-$0.31)
*Sex*		
K	0.78 (0.76,0.79)	$-$0.39 ($-$0.45,$-$0.34)
M	0.76 (0.75,0.77)	$-$0.26 ($-$0.31,$-$0.20)
*Country of origin*		
Denmar	0.77 (0.76,0.78)	$-$0.35 ($-$0.39,$-$0.31)
Not Denmark	0.73 (0.69,0.76)	$-$0.43 ($-$0.57,$-$0.30)
*Maternal education*		
Primary	0.68 (0.66,0.71)	$-$0.24 ($-$0.33,$-$0.15)
Upper secondary	0.78 (0.76,0.79)	$-$0.30 ($-$0.36,$-$0.24)
Bachelor’s	0.79 (0.78,0.81)	$-$0.32 ($-$0.39,$-$0.26)
Master’s or PhD	0.84 (0.82,0.87)	$-$0.30 ($-$0.41,$-$0.18)

### Grade point average (GPA)

Of the 444,793 children included in the analysis of school completion, a total of 410,540 attended the 9th grade exams and were included in the analysis of GPA. In the overall analysis of GPA, we found that adolescents with an SCD diagnosis in average had 0.36 lower GPA at exams in 9th grade compared to adolescents without an SCD diagnosis. These results persisted in the marginal analysis with interactions (Table2) and the expanded analysis including all covariates when comparing adolescents with same sex, country of origin, and maternal education. The estimated GPAs across all combinations of covariates are visualized in Fig. 2b. Notably, boys without an SCD diagnosis had lower GPAs (6.86 [95% CI 6.85,6.87]) than girls with an SCD (7.51 [95% CI 7.45, 7.56]) who in turn had lower GPAs than girls without SCD (7.90 [95% CI 7.89, 7.91]). Both non-Danish country of origin and lower maternal education were associated with lower GPAs as seen in Fig. 2b.

**Fig. 3:**
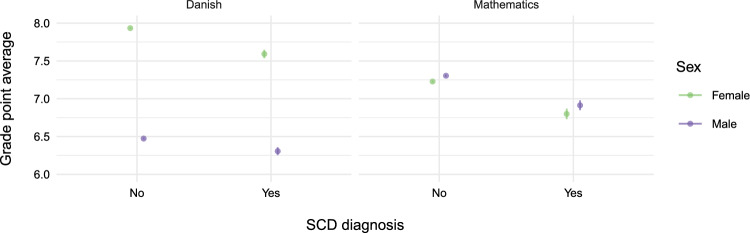
Estimated grade point average in Danish and mathematics for individuals with and without severe chronic disease (SCD) diagnosis adjusted for sex

In subject-specific GPA analyses (Fig. 3) boys with an SCD diagnosis had substantially lower GPA in Danish. However, there was no difference in the GPA of practical importance in mathematics between boys and girls. Similar results were found in analyses adjusted for country of origin and maternal education ("Appendix D").

### Danish national tests

**Fig. 4:**
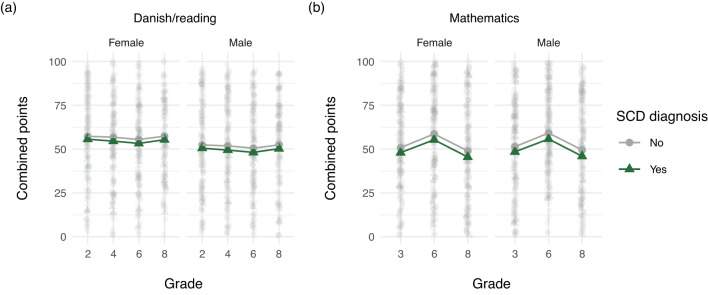
Estimated combined points from the Danish National Tests (DNT) in **a** the Danish/reading tests in grades 2, 4, 6, and 8, and in **b** the mathematics tests in grades 3, 6, and 8. Points adjusted for sex and severe chronic disease (SCD) diagnosis

In total, 155,520 children were included in the longitudinal analysis. The DNT analyses (Fig. 4) support the GPA findings for Danish exams (Fig. 2 and Fig. 3). In Danish/reading boys without an SCD diagnosis scored lower results than girls with an SCD, who in turn scored lower than girls without an SCD diagnosis. In mathematics, students with an SCD diagnosis had lower test results overall, however, there was no difference between boys and girls. Expanded analyses including all covariates ("Appendix A") showed similar patterns as previously described and these patterns were evident across all tested grade levels.

### Analyses by type of chronic disease

**Fig. 5:**
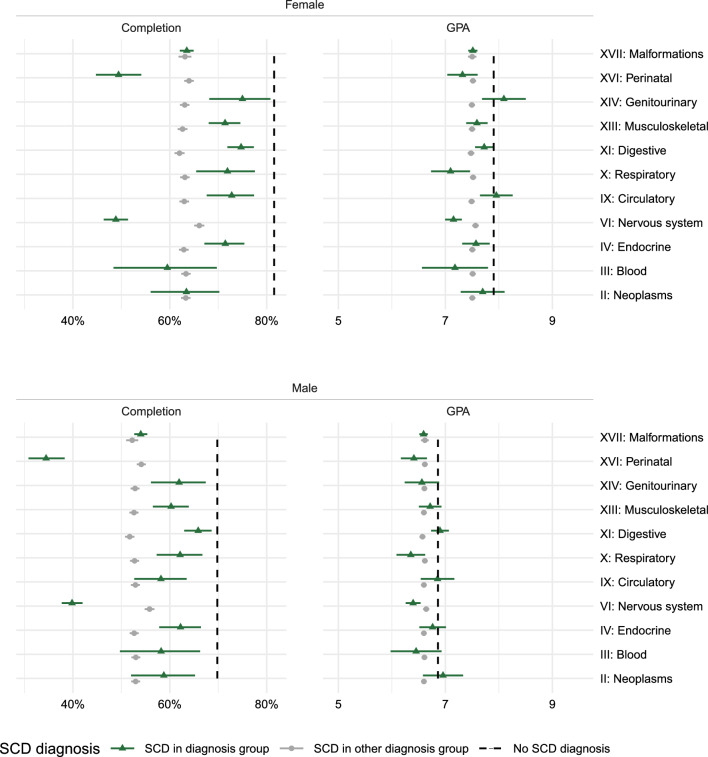
Sub-group analyses on ICD-10 chapters: estimated probabilities of completing 9th grade (left) and estimated grade point average (GPA; right) adjusted for severe chronic disease (SCD) diagnosis and sex

Analyses by type of chronic disease showed substantial variation in 9th grade completion probabilities and GPAs across diagnostic groups (Fig. 5). Children with neurological and perinatal SCD had markedly lower completion probabilities compared to other groups, followed by diseases of the blood, major congenital malformations and neoplasms. GPA analyses showed lower scores compared to peers for most organ systems-except for the digestive, circulatory, and genitourinary systems, and for neoplasms. Also, among boys, no significant GPA differences were observed for endocrine or blood disorders (Fig. 5).

### Sensitivity analyses of the landmarking approach

The cumulative incidence of SCD reached 9% by age 17 (compared to 4.7% at age 6), with higher incidence in boys (9.2%) compared to girls (8.8%) (Fig. 1). These differences may extend to the ways in which SCD affect educational outcomes prompting sensitivity analyses of the landmarking approach.

**Fig. 6:**
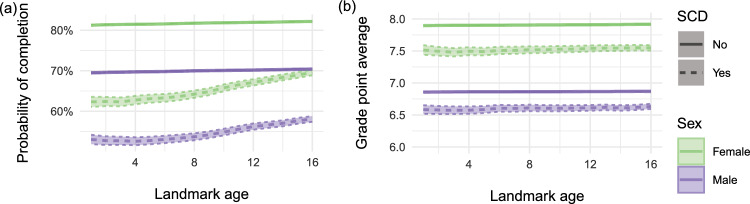
Estimated probability of **a** completing 9th grade and **b** estimated GPA in 9th grade based on different landmark ages

For the analyses of completion of lower secondary school, the results are shown in Fig. 6a and indicate a similar effect of (landmark) age of diagnosis across all combinations of covariates, namely that the later the diagnosis the higher the probability of completing 9th grade. For the analyses of grade point averages, the results are shown in Fig. 6b and indicate no notable effect of age of diagnosis across combinations of covariates.

While children diagnosed with certain SCDs before school entry, in particular neurological and perinatal SCD, had markedly lower completion probabilities compared to other groups (Fig. 5), sensitivity analyses showed that among children diagnosed after school entry ("Appendix D") completion rates were more similar across diagnoses, although consistently lower in boys than in girls.

## Discussion

This nationwide cohort study provides robust evidence that Danish children expected to complete 9th grade between 2017 and 2023 and diagnosed with severe chronic disease (SCD) face notably poorer educational outcomes compared to their peers. We observed markedly lower probabilities of completing lower secondary school and reduced grade point averages (GPAs) at the 9th-grade level among adolescents with SCD. These disparities persisted after adjusting for sex, country of origin, and maternal education level, underscoring the independent association between chronic illness and school performance.

Our findings are consistent with prior research indicating that children with chronic health conditions are at elevated risk of educational disadvantage [10, 11, 17]. However, our study contributes novel insights by using a large, population-based dataset from a country with universal access to healthcare and education. This context reduces possible confounding due to differential access to health or educational services and highlights that significant disparities in school performance persist even in welfare settings.

The variation in GPA across diagnostic groups and school subjects is notable. Children with SCD exhibited lower performance in Danish compared to mathematics, suggesting that performance in language-based subjects might be more sensitive to disruptions caused by illness or treatment. Interestingly, our findings also revealed important gender differences: boys had lower GPAs than girls, regardless of SCD status. In fact, boys without SCD performed worse than girls with SCD. This finding was subject-specific-pronounced in Danish but not in mathematics. These findings align with previous literature showing gender differences in academic outcomes in the general population and suggest that boys in general and boys with SCD in particular may be vulnerable to falling behind. Our results from the Danish National Tests (DNT) supported these findings and further revealed that the pattern was evident across all tested grade levels, indicating that academic disadvantages associated with SCD are persistent throughout the school years.

The relationship between maternal education, country of origin and academic outcomes further emphasizes the role of socioeconomic context. Our results present groups of children (based on their combination of covariates) who may be particularly vulnerable to poor educational outcomes not necessarily due to SCD. However, children of mothers with lower education experienced larger performance gaps associated with SCD, supporting previous findings that family background can amplify the impact of health-related challenges [16, 18].

Interestingly, our sensitivity analyses using a landmarking approach showed consistent effects of SCD on GPA regardless of the age at ascertainment of diagnosis suggesting that once students remain engaged through to the final exams, their performance may be less dependent on when the illness was diagnosed. However, age of diagnosis was shown to clearly impact school completion, as children diagnosed earlier were less likely to complete 9th grade.

The impact of SCD on school completion and GPA is also influenced by the underlying disorder. The definition of SCD applied in this study encompassed a heterogeneous group of conditions, some potentially curable, such as neoplasms, while others persist throughout childhood. Consistent with previous literature, we found that children with neurological conditions (e.g. epilepsy and cerebral palsy) and perinatal complications (e.g. asphyxia) had the lowest rates of 9th grade completion [11, 35]. These conditions are strongly linked to cognitive impairments, executive dysfunction, and increased need for special education support, which likely contribute to disengagement from school and reduced capacity to follow mainstream educational pathways. For those able to attend the regular 9th grade exams, GPAs were, however, comparable to peers with other types of SCD. In our study, all diagnostic groups were associated with lower completion rates of secondary school, and most with moderately lower GPAs. Previous studies have primarily focused on single conditions rather than broader diagnostic categories and have shown that impaired school performance is observed across a wide range of severe somatic disorders but not consistently for common childhood disorders including asthma, diabetes and inflammatory bowel disorders [11]. The present national cohort study supports and extend this literature by demonstrating that, even within a universal welfare context, children and adolescents with SCD–especially individuals experiencing perinatal diseases or neurological involvement–experience lower educational attainment. These findings reinforce the need for early identification and cross-sectoral support for vulnerable groups beyond the most frequently studied diagnostic categories.

Our findings are consistent with international research indicating that children with chronic health conditions are at elevated risk of educational disadvantage [10, 11, 17, 20]. Although direct comparison of effect sizes is limited by differences in study design and educational systems, our estimate of a $\sim 10-20\%$ lower probability of completing lower secondary education among children with SCD is broadly in line with Finnish population-based data reporting a relative risk of 1.27 for dropout by age 17 [11]. US data similarly suggest increased odds of not completing high school among adolescents with chronic medical conditions, though effect estimates are somewhat higher and measured at an older age (odds ratio 1.47, [20]).

The selection of covariates in our analyses was informed by previous literature and guided by conceptual frameworks linking chronic disease, socioeconomic context, and educational outcomes. We included sex, country of origin, and maternal educational level as covariates, given their well-documented associations with academic performance and health disparities [18, 19]. Sex differences in educational achievement are consistently observed, with boys often under performing relative to girls across a range of outcomes. Maternal educational level is a widely used proxy for family socioeconomic status and has been shown to be associated with children’s cognitive development, access to learning resources, and parental engagement with schools [36]. Country of origin was included to account for potential disparities related to migration background and language proficiency. By adjusting for these variables, we aimed to better isolate the association between SCD and school performance and to identify subgroups who may face compounding vulnerabilities. Nevertheless, we acknowledge that other relevant factors, such as family structure, school-level characteristics, or the severity of illness, were not available in our dataset and may also influence educational trajectories.

Our study has several strengths, including a large sample size, national coverage, and linkage of administrative data on health and education. Furthermore, our choice of statistical methods aimed to provide robust and interpretable estimates. To address potential bias due to the time-dependent nature of exposure (i.e., the timing of SCD diagnosis), we employed a landmark approach, setting the exposure status at a fixed time point (the year of turning six). This design avoids immortal time bias and ensures that exposure precedes outcome measurement, a critical aspect of valid inference in longitudinal studies. We further conducted sensitivity analyses across a range of landmark ages to evaluate the robustness of our findings. For repeated test scores in the Danish National Test Program, we used mixed-effects models to account for within-subject correlations and varying numbers of observations per individual.

Nonetheless, several limitations merit consideration. First, while we used a validated classification of SCD based on ICD-10 codes, misclassification of exposure or under reporting may still occur. Second, although we adjusted for key sociodemographic covariates, factors such as school-level support, parental involvement, or illness severity may contribute to unmeasured confounding. Third, we acknowledge the challenge regarding proxy measures of socioeconomic status. While maternal education in other studies has been found to be the most important socioeconomic predictor of child achievements [36], and a Danish study found that lower maternal education was associated with adverse perinatal outcomes [37], maternal education is not the only socioeconomic factor of importance. Fourth, the exclusion of children attending special needs schools or being exempt from exams may have led to underestimation of the true academic impact of SCD. Finally, it is important to note that we did not formally account for censoring due to death or emigration after the landmark age. These individuals were excluded from the analyses. However, the proportion of censored individuals was low, and likely had minimal impact on the overall estimates. Future studies could address this aspect further.

Our findings underscore the importance of early and sustained and social support tailored to both health and socioeconomic vulnerability. Schools, clinicians, and policymakers should work collaboratively to identify at-risk students and implement tailored interventions that account for both medical and socio-environmental needs.

Future research should explore specific disease trajectories, treatment methods, and protective factors that may mitigate risk of poor school performance. Additionally, qualitative studies may help elucidate the lived experiences of these children and their families, offering insights into barriers and enablers of educational success not captured by register data.

## Appendix A: Supplementary material on the Danish National test program

### A.1: Outcome

The Danish National Test Program (DNT) was introduced in 2010 to enhance nationwide testing and assessment methods in public schools in Denmark. The primary goal was to provide teachers with a clearer understanding of students’ academic progress throughout their school years, offering a comparison to the broader academic performance of Danish students. Previously, the final 9th-grade served as the main indicator of student performance. The national tests aim to foster stronger collaboration between schools and families by providing more continuous and detailed insights into student achievement. Test scores in reading (Danish) and mathematics from DNT were obtained for children aged 8–16 attending public schools in Denmark from 2010 to 2020. Children enrolled in Danish public schools are required to take ten national tests during compulsory schooling; a reading test every second year from the second grade, a mathematics test in grades 3, 6, and 8 (mathematics in grade 8 was introduced in 2018), and other subject-specific tests in grades 7 and 8. Furthermore, teachers may opt to test students twice in the grade level prior to/after the intended level on a voluntary basis. Each test simultaneously assesses three cognitive domains, called profile areas. For example, the reading tests assess language comprehension, decoding, and reading comprehension, while the mathematics tests assess numbers and algebra, geometry, and applied mathematics. Different scales are used for calculation and for communication of the test results. We used the percentile scale (points) where test results were communicated using the norm-based percentile scale (range 1–100, higher scores indicate better performance). The score reflects the student’s performance as a percentile according to the nationwide score distribution on the same test in the 2010 pilot test consisting of 15,000–22,000 students. We included two co- secondary outcomes to evaluate the school performance in children with and without SCD over time. We examined the results from the Danish/reading tests in grades 2, 4, 6, and 8 and the results from the mathematics tests in grades 3, 6, and 8. To measure the student’s overall skills, we used a standardized test score measure combining student skill levels in all three cognitive domains.

### A.2: Methods

The statistical analyses were based on mixed-effects linear regression models with the combining points in all three cognitive domains the tests as the outcome. We used a clinical diagnosis of SCD from birth to 31 July the year of taking the tests as the time-dependent exposure. The variation in the test results was associated with students and with the schools. We were interested in the variation due to students and in assessing the potency of the schools after accounting for this variation. Thus, we used random effects for the student factor. We also used random effects for the school factor because we were more interested in the school-to-school variability than in the potency of a particular school. In this study with multiple so-called grouping factors (student and school), the configuration of the factors was neither nested nor completely crossed; students with test results in multiple years could have been associated with different schools for the different years, that is, the student factors cannot be nested within the school factor. Conversely, student and school factors were not completely crossed. To have completely crossing of the student and school factors it would be necessary for each student to be observed with each school, which (would be unusual and) was not the case. That is, we had so-called partially crossed grouping factors for the random effects. We fitted two versions of the mixed effects models: one overall model of the exposures effect on the outcome adjusted for sex and school grade and one model further adjusted for country of origin and maternal education.

### A.3: Results

Characteristics of covariates and outcomes across the study populations used in the two co-secondary analyses are provided in Tables 3 and 4.

**Table 3:** Characteristics of the study populations used for the analyses of the Danish national tests (DNTs) in Danish/reading in grades 2, 4, 6, and 8

Characteristic	2	4	6	8
*Sex*				
Male	73,190 (50.3%)	72,121 (50.4%)	70,347 (50.5%)	64,749 (50.9%)
Female	72,306 (49.7%)	71,085 (49.6%)	68,860 (49.5%)	62,444 (49.1%)
*Country of origin*				
Denmark	12,522 (8.6%)	130,733 (91.3%)	12,492 (9.0%)	12,247 (9.6%)
Not Denmark	132,974 (91.4%)	12,473 (8.7%)	126,715 (91.0%)	114,946 (90.4%)
*Maternal education*				
Upper secondary	1,785 (1.2%)	20,070 (14.0%)	57,263 (41.1%)	14,424 (11.3%)
Primary	60,051 (41.3%)	15,937 (11.1%)	1,763 (1.3%)	16,279 (12.8%)
Master’s or PhD	46,515 (32.0%)	46,588 (32.5%)	15,530 (11.2%)	52,413 (41.2%)
Bachelor’s	15,880(10.9%)	58,824 (41.1%)	18,914 (13.6%)	42,409 (33.3%)
Unknown	21,265 (14.6%)	1,787 (1.2%)	45,737 (32.9%)	1,668 (1.3%)
*Severe chronic disease*				
No	6,763 (4.6%)	135,593 (94.7%)	130,910 (94.0%)	118,886 (93.5%)
Yes	138,733 (95.4%)	7,613 (5.3%)	8,297 (6.0%)	8,307 (6.5%)
*Combined points*				
Mean (SD)	56.7 (24.8)	56.1 (24.7)	54.7 (23.5)	56.5 (22.4)

**Table 4:** Characteristics of the study populations used for the analyses of the Danish national tests (DNTs) in mathematics in grades 3, 6, and 8

Characteristic	3	6	8
*Sex*			
M	71,465 (49.6%)	70,254 (50.6%)	46,859 (52.2%)
K	72,500 (50.4%)	68,709 (49.4%)	42,928 (47.8%)
*Country of origin*			
Denmark	12,336 (8.6%)	12,479 (9.0%)	81,113 (90.3%)
Not Denmark	131,629 (91.4%)	126,484 (91.0%)	8,674 (9.7%)
*Maternal education*			
Upper secondary	46,495 (32.3%)	1,755 (1.3%)	1,241 (1.4%)
Primary	15,928 (11.1%)	15,528 (11.2%)	10,398 (11.6%)
Master’s or PhD	59,301 (41.2%)	57,178 (41.1%)	11,476 (12.8%)
Bachelor’s	1,765 (1.2%)	18,806 (13.5%)	36,605 (40.8%)
Unknown	20,476 (14.2%)	45,696 (32.9%)	30,067 (33.5%)
*Severe chronic disease*			
No	136,842 (95.1%)	8,251 (5.9%)	83,773 (93.3%)
Yes	7,123 (4.9%)	130,712 (94.1%)	6,014 (6.7%)
*Combined points*			
Mean (SD)	53.1 (25.3)	60.6 (24.4)	50.5 (26.6)

Of the 194,371 children registered in the MBR as live born in Denmark between 1 January 2002 and 31 December 2004, 166,642 (85.7%) adolescents completed one or more tests in reading (Danish) or mathematics from the DNT. In total, 155,520 (80%) children were included in the longitudinal study since 1015 (0.5%) children who died and 4820 (2.5%) children who migrated before 31 July the year of the first test (in second grade) were excluded. Furthermore, 150 (0.1%) children with an unknown country of origin were excluded, as were 503 (0.3%) children whose mother died before 31 July the year of the first test and 405 (0.2%) students who took a test on a level different to the one assigned their grade. Overall, 76,697 (49.3%) were female, 141,369 (90.9%) were of Danish origin, and 63,496 (40.8%) were born to mothers with the highest educational attainment of upper secondary school, the most common highest educational level among the mothers at the time their child turned eight. Among the children and adolescents who were included in the study 119 (0.1%) died while 1, 915 (1.2%) migrated before 30 July the year of turning sixteen. The mother of 499 (0.3%) children died during the study period. Other likely reasons for children not participating in the tests were because they attended independent schools (friskoler), attended public schools but were missing a result, or attended a special needs school.

In the expanded analysis of combined points from the DNT including all covariates, we found that students with an SCD diagnosis had lower test results when comparing students with same sex, country of origin, and maternal education. This was evident in both Danish/reading and i mathematics. The estimated combined points across all combinations of covariates are visualized in Fig. 7a for the Danish/reading tests and in Fig. 7b for the mathematics tests. When comparing students with same country of origin and maternal education we further observed that boys without an SCD diagnosis had lower combined points in Danish/reading than girls with an SCD, who in turn had lower combined points than girls without an SCD diagnosis. Furthermore, we found that lower maternal education was associated with lower test results in both subjects. The analyses showed that these patterns were evident across all tested grade levels.

Fig. 7 Estimated combined points from the Danish NationalTests (DNT) in **a** the Danish/reading tests in grades 2, 4, 6, and 8, and in **b** the mathematics tests in grades 3, 6, and 8. Points adjusted for sex, severe chronic disease (SCD) diagnosis, country of origin, and maternal education

## Figures and Tables

**Fig. 8:**
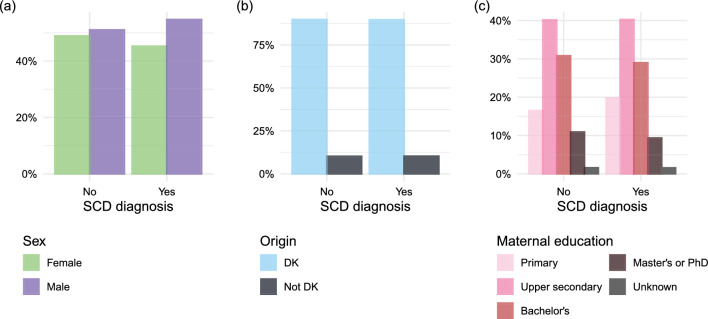
Distribution of **a** sex, **b** country of origin, and **c** maternal education. Results were stratified by severe chronic disease (SCD) diagnosis

**Fig. 9:**
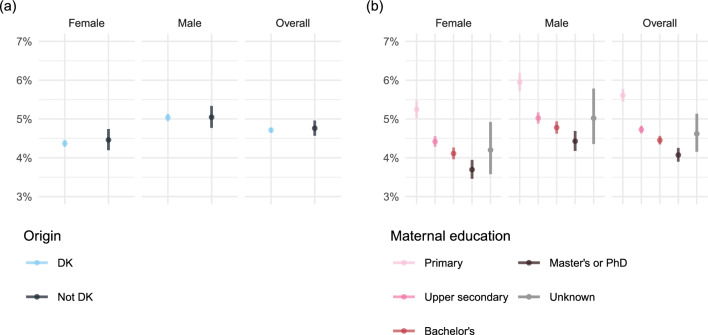
Estimated probability of severe chronic disease (SCD) diagnosis by **a** country of origin and **b** maternal education

**Fig. 10:**
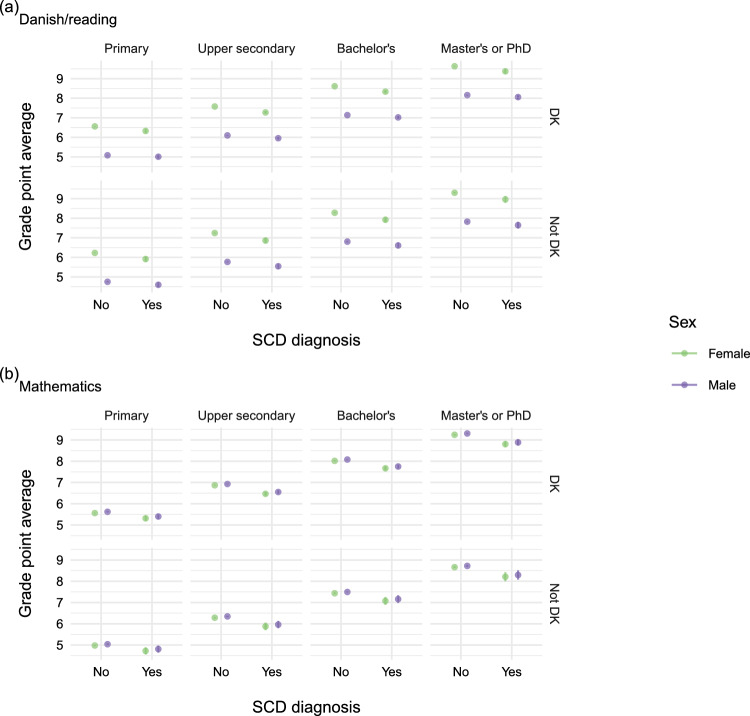
Estimated grade point average in Danish **a** and mathematics **b** for individuals with and without severe chronic disease (SCD) diagnosis adjusted for sex, country of origin, and maternal educational level

**Fig. 11:**
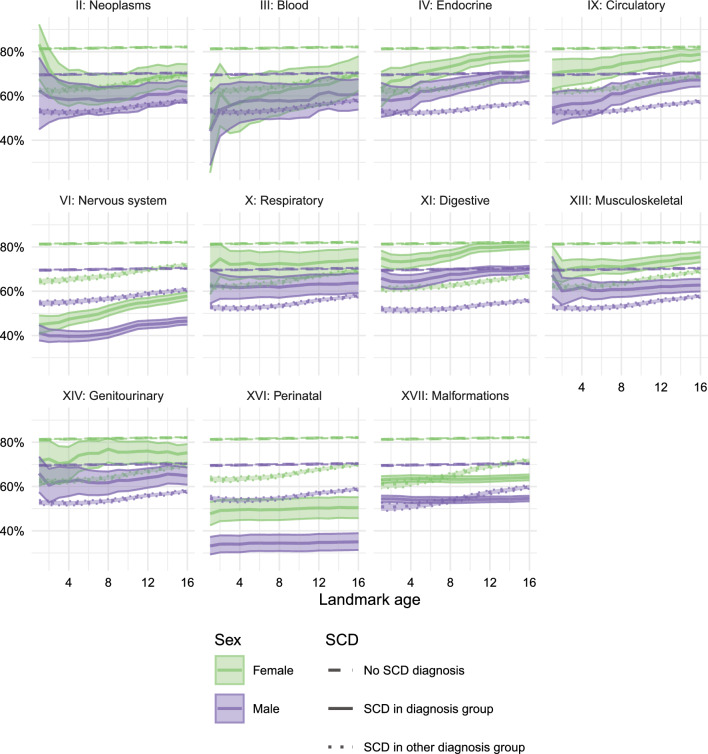
Sub-group sensitivity analyses on ICD-10 chapters: estimated probabilities of completing 9th grade based on different landmark ages adjusted for severe chronic disease (SCD) diagnosis and sex.

## Data Availability

Not applicable.

## Appendix B: Definition of severe chronic disease

SCD in children was defined using ICD-10 codes (Table 5) and categorized based on the chapters of the ICD?10.

**Table 5:** Definition of severe chronic disease by ICD-10 codes, by chapter

Chapter no. and name	ICD-10 codes
II: Neoplasms	C00.0–C99.1
III: Blood	D61.0; D61.3; D61.8–D61.9; D76.2; D80.0–D82.9
IV: Endocrine	E10; E25; E70.0–E73.0; E74.0–E84.9
VI: Neurology	G12; G31.0; G31.8–G31.9; G37.0–G37.9; G40; G60; G70.2; G71.0–G71.3; G73.6; G80; G81.1; G82.1; G82.4; G91; G94.1
IX: Circulatory	I12; I27.0–I27.9; I30.0–I52.8
X: Respiratory	J44.8; J84
XI: Digestive	K21; K50.0–K51.9; K70.0–K77.8; K90
XIII: Musculoskeletal	M30.0–M35.9
XIV: Genitourinary	N03–N05; N07; N13; N18.0–N19.9; N25.0–N27.9
XVI: Perinatal	P27; P57.0–P57.9; P91.0–P91.2; P94.1–P94.9
XVII: Malformations	Q01–Q07; Q20.0–Q26.9; Q30.0–Q32.4; Q33.0–Q33.9; Q34.0–Q37.9; Q39.0–Q45.3; Q60.0–Q64.9; Q79.0; Q79.2–Q79.3; Q86.0; Q87; Q90.0–Q99.9

## Appendix C: Supplementary methods

### C.1: Covariates

Maternal education was defined as the highest level of formal education completed by the mother at the primary landmark and was categorized according to the UNESCO international standard classification of education [32] and divided into four categories: primary education (including pre-school educations, lower secondary education and preparatory courses), secondary education (including upper secondary education, Danish language courses at language centers, vocational education and training [VET], qualifying educational programs, and labor market educations [AMU]), bachelor’s degree (including short cycle higher education, vocational bachelor’s educations, and bachelor’s programs), and master’s or PhD programs (including master’s programs and PhD programs). Finally, we included a category including no education or unknown.

## Appendix D: Supplementary results

### D.1: Population

Among the 444,793 children included in the first co-primary analysis a total of 410,540 attended the exams and were thus included in the second co-primary analysis of the average mark at exams in grade 9. Overall, 202, 167 (49.2%) were female, 373, 301 (90.9%) had a mother and/or father whose country of origin was Denmark, and 166, 795 (40.6%) were born to mothers with highest educational attainment of upper secondary school, the most common highest educational level among the mothers at the time their child turned six.

### D.2: Confounding

The covariates sex, country of origin, and maternal education were fairly balanced in the exposed (SCD diagnosis by the primary landmark) and non-exposed (no SCD diagnosis by the primary landmark) groups (Fig. 8). However, exploratory analyses of pairwise associations showed that sex and maternal education were associated with an SCD diagnosis while country of origin was not (Fig. 9 contain estimates of the pairwise associations). Specifically, boys had higher probability of having an SCD diagnosis (5% [95% CI 4.9, 5.1]) compared to girls (4.4% [95% CI 4.3, 4.5]) and children whose maternal education was lower had higher probability of having an SCD diagnosis (primary school: 5.6% [95% CI 5.4, 5.8], upper secondary school: 4.7% [95% CI 4.6, 4.8], Bachelor’s degree: 4.5% [95% CI 4.3, 4.6], Master’s or PhD degree: 4.1% [95% CI 3.9, 4.3]). Furthermore, children whose maternal education was unknown had a probability of 4.6% (95% CI 4.2, 5.1) of having an SCD diagnosis.

### D.3: Crude estimates of the probability of completing grade 9

Exploratory analyses of pairwise associations between the probability of completing 9th grade and the covariates of interest showed that sex, country of origin, SCD, and maternal education were associated with the probability of completing 9th grade. Specifically, they showed that boys had lower probability of completing 9th grade (68.9% [95% CI 68.7, 69.1]) compared to girls (80.7% [95% CI 80.5, 80.9]); Adolescents whose country of origin was not Denmark had lower probability of completing 9th grade (61.1% [95% CI 60.6, 61.5]) compared to adolescents whose country of origin was Denmark (76.2% [95% CI 76.1, 76.3]); Adolescents with an SCD diagnosis had lower probability of completing 9th grade (57.6% [95% CI 57, 58.3]) compared to adolescents without an SCD diagnosis (75.5% [95% CI 75.4, 75.6]); Adolescents whose maternal education was lower had lower probability of completing 9th grade (primary school: 58% [95% CI 57.7, 58.4], upper secondary school: 74.8% [95% CI 74.6, 75], Bachelor’s degree: 81.4% [95% CI 81.2, 81.6], Master’s or PhD degree: 84.2% [95% CI 83.9, 84.5]).

### D.4: Crude estimates of grade point averages

Exploratory analyses of pairwise associations showed that sex, country of origin, SCD, and maternal education were associated with the average mark at exams in 9th grade for adolescents completing 9th grade. Specifically, they showed that boys had lower average mark at exams in 9th grade (6.7 [95% CI 6.7, 6.7]) compared to girls (7.7 [95% CI 7.7, 7.8]); Adolescents whose country of origin was not Denmark had lower average mark at exams in 9th grade (6.2 [95% CI 6.2, 6.2]) compared to adolescents whose country of origin was Denmark (7.3 [95% CI 7.3, 7.3]); Adolescents with an SCD diagnosis had lower average mark at exams in 9thgrade (6.9 [95% CI 6.8, 6.9]) compared to adolescents without an SCD diagnosis (7.2 [95% CI 7.2, 7.2]); Adolescents whose maternal education was lower had lower average mark at exams in 9th grade (primary school: 5.6 [95% CI 5.6, 5.7], upper secondary school: 6.9 [95% CI 6.8, 6.9], Bachelor’s degree: 7.9 [95% CI 7.9, 7.9], Master’s or PhD degree: 9 [95% CI 8.9, 9]).

### D.5: Subject specific results

The expanded analyses of GPA further adjusted for country of origin and maternal education showed that students with an SCD diagnosis had lower GPA than their peers in both Danish (Fig. 10a) and mathematics (Fig. 10b). Moreover, the analyses showed a difference between sexes in Danish, where girls performed markedly better than boys across all combinations of country of origin and maternal education. In contrast, boys obtained slightly higher GPA than girls in mathematics. Lower maternal education was associated with lower GPA in both subjects as was country of origin other than Denmark.

### D.6: Sensitivity analysis

Results of the sensitivity analyses of the landmarking approach by type of chronic disease are shown in Fig. 11
