# Supplementary figures and images for: Association between chronic diseases in childhood and subsequent educational achievement: a Danish register-based cohort study
Source: Eur J Epidemiol. 2025 Oct 14;41(1):89–105. doi: 10.1007/s10654-025-01315-9 (PMC12881040; doi:10.1007/s10654-025-01315-9)

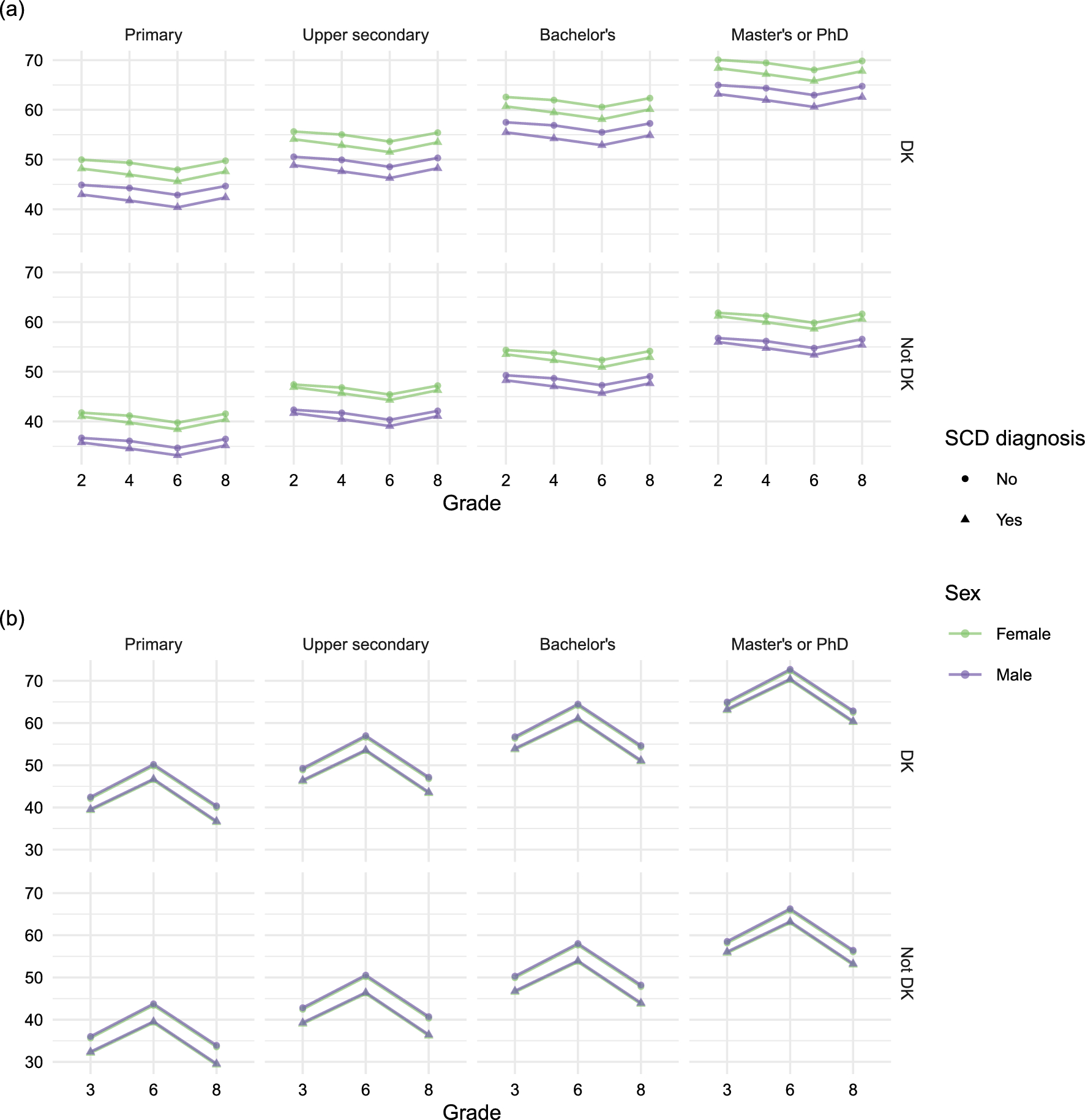

Supplement: Supplementary file 1 — Estimated combined points from the Danish NationalTests (DNT) in a the Danish/reading tests in grades 2, 4, 6, and 8, and in b the mathematics tests in grades 3, 6, and 8. Points adjusted for sex, severe chronic disease (SCD) diagnosis, country of origin, and maternal education [file 10654_2025_1315_Fig7_HTML.png]
